# Isolation and Characterization of Barley (*Hordeum vulgare*) Extracellular Vesicles to Assess Their Role in RNA Spray-Based Crop Protection

**DOI:** 10.3390/ijms22137212

**Published:** 2021-07-05

**Authors:** Timo Schlemmer, Patrick Barth, Lisa Weipert, Christian Preußer, Martin Hardt, Anna Möbus, Tobias Busche, Aline Koch

**Affiliations:** 1 Institute of Phytopathology, Centre for BioSystems, Land Use and Nutrition, Justus Liebig University, Heinrich-Buff-Ring 26, 35392 Giessen, Germany; timo.schlemmer@agrar.uni-giessen.de (T.S.); weipert@dib.org (L.W.); 2 Institute of Phytomedicine, University of Hohenheim, Otto-Sander-Strasse 5, 70599 Stuttgart, Germany; 3 Institute of Bioinformatics and Systems Biology, Justus Liebig University, Heinrich-Buff-Ring 58, 35392 Giessen, Germany; patrick.barth@computational.bio.uni-giessen.de; 4 Institute for Tumor Immunology, Center for Tumor Biology and Immunology (ZTI), Philipps University, Hans-Meerwein Strasse 3, 35032 Marburg, Germany; preusserc@staff.uni-marburg.de; 5 Imaging Unit, Biomedical Research Centre Seltersberg (BFS), Justus Liebig University, Schubertstrasse 81, 35392 Giessen, Germany; Martin.Hardt@bfs.uni-giessen.de (M.H.); anna.moebus@bfs.uni-giessen.de (A.M.); 6 Centre for Biotechnology—CeBiTec, Bielefeld University, Universitätsstrasse 25, 33615 Bielefeld, Germany; tbusche@cebitec.uni-bielefeld.de

**Keywords:** plant EV, extracellular vesicles, RNA interference, RNAi, siRNA, dsRNA, RNA spray, barley, *Fusarium graminearum*

## Abstract

The demonstration that spray-induced gene silencing (SIGS) can confer strong disease resistance, bypassing the laborious and time-consuming transgenic expression of double-stranded (ds)RNA to induce the gene silencing of pathogenic targets, was ground-breaking. However, future field applications will require fundamental mechanistic knowledge of dsRNA uptake, processing, and transfer. There is increasing evidence that extracellular vesicles (EVs) mediate the transfer of transgene-derived small interfering (si)RNAs in host-induced gene silencing (HIGS) applications. In this study, we establish a protocol for barley EV isolation and assess the possibilities for EVs regarding the translocation of sprayed dsRNA from barley (*Hordeum vulgare*) to its interacting fungal pathogens. We found barley EVs that were 156 nm in size, containing predominantly 21 and 19 nucleotide (nts) siRNAs, starting with a 5′-terminal Adenine. Although a direct comparison of the RNA cargo between HIGS and SIGS EV isolates is improper given their underlying mechanistic differences, we identified sequence-identical siRNAs in both systems. Overall, the number of siRNAs isolated from the EVs of dsRNA-sprayed barley plants with sequence complementarity to the sprayed dsRNA precursor was low. However, whether these few siRNAs are sufficient to induce the SIGS of pathogenic target genes requires further research. Taken together, our results raise the possibility that EVs may not be mandatory for the spray-delivered siRNA uptake and induction of SIGS.

## 1. Introduction

RNAi-based plant protection strategies represent powerful tools to address the goals of the European “farm to fork” strategy in order to reduce the usage of pesticides by approximately 50% by 2030. As an alternative to conventional pesticides, RNAi-based plant protection holds enormous potential to prevent further drastic losses of biodiversity. Over the last two decades, more than 170 studies have demonstrated the feasibility of controlling agronomically and horticulturally relevant plant diseases by utilizing the transgenic expression (host-induced gene silencing (HIGS) [1]) and exogenous application (spray-induced gene silencing (SIGS) [2]) of double-stranded RNA (dsRNA) to trigger the post-transcriptional gene silencing of target genes in various plant pathogens and pests [3]. In addition to the academic proof-of-concept of numerous pathosystems, RNAi technology has further advanced to enable lab-to-field transitions (e.g., costs, risk assessments, formulations, and regulations). Despite such significant achievements, we still lack a mechanistic understanding of these technologies. For example, the mechanisms underlying the transfer and uptake of transgene- or spray-derived RNAs during plant–fungal interactions remain ill-defined, yet they play a pivotal role in determining the efficacy and specificity of RNAi-based plant protection. We predict that closing these gaps in the knowledge will facilitate the development of novel integrative concepts, precise risk assessments, and tailor-made RNAi therapies for plant diseases. To this end, we assessed the role of extracellular vesicles (EVs) in the transfer of SIGS-inducing RNAs.

Recent data suggest that, analogous to the role of mammalian exosomes in cell-to-cell communication, fungi rely on a bidirectional sRNA transport system mediated by EVs [4]. Supporting this, we recently found that EVs purified from *Arabidopsis thaliana* leaf extracts and apoplastic fluids contain transgene-derived small interfering RNAs (siRNAs) [5 preprint]. Furthermore, RNA sequencing (RNA-seq) analysis reveals that EVs from plants expressing CYP3RNA, a 791 nucleotide (nts) long dsRNA originally designed to target the three *CYP51* genes of the fungal pathogen *Fusarium graminearum* (*Fg*), contain CYP3RNA-derived siRNAs [5]. Notably, the EVs’ cargo retained the same CYP3RNA-derived siRNA profile as that of the respective leaf extracts, suggesting that there was no selective uptake of specific artificial sRNAs into EVs. Moreover, mutants of the endosomal sorting complex required for transport-III (ESCRT-III) were impaired in HIGS, and EVs were free of CYP3RNA-derived siRNAs [5]. The latter serves as further indication that endosomal vesicle trafficking supports the transfer of transgene-derived siRNAs between donor host cells and recipient fungal cells. Although the number of EV-contained siRNAs was low, we lack information on the minimum concentration of siRNAs required inside an EV to induce HIGS. Notably, we demonstrated previously that *Fg* can take up long, unprocessed dsRNA that is processed by its own RNAi [6,7]. This finding, which may explain why we observed greater silencing efficacy in the fungal target genes [8], indicates that fungi, like insects, seem to respond more efficiently to dsRNA than to siRNA. Feeding on dead plant tissue, necrotrophic fungi may take up topically applied dsRNA or dsRNA delivered to the xylem [6]. Consequently, we speculate that the role of EVs in mediating siRNA uptake is minor in the SIGS–*Fg*–barley system. In the present study, we isolated, for the first time, EVs from dsRNA-sprayed barley leaves and analyzed their RNA cargo to verify whether barley EVs contain dsRNA spray-derived siRNAs. In addition, we compared our RNA-seq results with existing datasets of EVs isolated from CYP3RNA-expressing *Arabidopsis* plants to seek sequence similarities between siRNAs in EVs isolated using HIGS and SIGS strategies, respectively.

## 2. Results and Discussion

**Figure 1:**
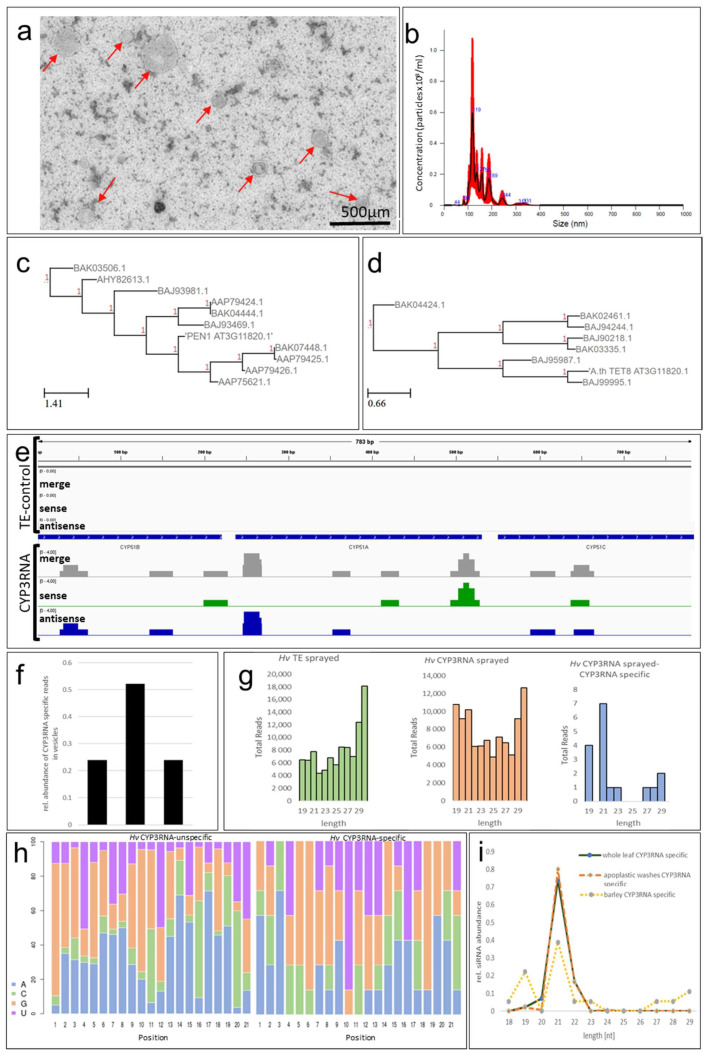
(**a**) Barley EVs were negatively stained onto copper formvar meshes using 2% uranyl acetate. (**b**) Next, 5 µL of purified EVs was diluted up to a volume of 500 µL. The vesicle suspension was loaded onto a Nanosight NS300 (Malvern Panalytical). Five measurements were performed at 25 °C, and size, concentration prediction, and statistical analyses were performed using the NTA 3.2 Dev Build 3.2.16 software. (**c**,**d**) *Arabidopsis thaliana* PEN1 (AT3G11820) and TET8 (AT2G23810) paralogs of *Hordeum vulgare* subsp. vulgare (*Hv*) were predicted by the NCBI’s protein BLAST service (Available online: https://blast.ncbi.nlm.nih.gov/Blast.cgi (accessed on 14 June 2021)) and visualized using the ete view tool. Available online: http://etetoolkit.org (accessed on 23 June 2021). (**e**) RNA was isolated from mock and dsRNA-treated barley leaves. Indexed sRNA libraries were pooled and sequenced on the Illumina MiSeq Platform (1 × 36 SE). The readings were then mapped onto the CYP3RNA sequence. (**f**) The relative abundance of reads aligned to each CYP3RNA fragment (*CYP51A*, *CYP51B*, *CYP51C*) were calculated and (**g**) reads were sorted based on their size. (**h**) The nucleotide distribution for every position was counted for the 21 nts long siRNAs of all barley siRNAs (left) and siRNAs with perfect complementarity towards the CYP3RNA precursor (right). (**i**) The relative abundances of siRNA with different lengths from barley EVs were compared with relative siRNA abundances from *Arabidopsis* EVs purified from apoplastic washes and *Arabidopsis* vesicles isolated by whole-leaf vesicle purification.

To study whether barley (*Hordeum vulgare*) EVs contain dsRNA spray-derived siRNAs, we established a protocol for EV isolation from barley leaves by adjusting the EV isolation protocol we previously adopted for *Arabidopsis* preparation [5]. Accordingly, barley leaves were sprayed with CYP3RNA as described in [6] and the lower unsprayed leaf segments were harvested and freshly cut at both ends immediately before being submerged in the vesicle isolation buffer. The duration of vacuum infiltration was increased to four minutes and was repeated three times to fully infiltrate the barley leaves. In comparison, *Arabidopsis* leaves required only one minute per round to achieve full leaf infiltration. To harvest the apoplastic fluid, the centrifugation speed was adapted from 700× *g* to 1000× *g* for 20 min at 4 °C. Purified barley EVs exhibited a highly diverse size distribution with a mean size of 156 +/− 12.2 nm, which is slightly larger than the mean size of EVs isolated from *Arabidopsis* (139 +/− 7.7 nm [5,9], Figure 1a,b), but still fits well within the standard 50–300 nm range for plant EVs [4,10]. Transmission electron microscopy (TEM) revealed no obvious differences in electron density for barley EVs compared to previously described *Arabidopsis* EVs [5] (Figure 1a), indicating the similar appearance of EVs isolated from the two different plant species. Notably, nanoparticle trafficking analysis (NTA) and TEM displayed a strong heterogenicity of size among barley EVs compared to *Arabidopsis* EVs. NTA revealed several peaks between 100 and 250 nm, which were confirmed by TEM-based size measurements (Figure 1a,b). However, further mechanistic research is required to confirm the differences in EV biogenesis between monocot and dicot plant species that might explain the heterogenicity of EV populations. To the best of our knowledge, this is the first report on EVs isolated from barley leaves. Thus, we currently lack an EV marker for immunodetection, which is necessary to prove the EVs’ origin. For *Arabidopsis* EVs, syntaxin PENETRATION1 (PEN1) [9] and TETRASPANIN8 (TET8) [11] are the referenced EV markers. Currently, the limited information on EV markers in *Arabidopsis* as the plant model organism further impedes efforts to identify possible barley EV markers. Based on the amino acid similarity, we located 10 homologs for PEN1 and seven homologs for TET8 in barley (Figure 1c,d). However, whether the identified PEN1 and TET8 homologs represent valid barley EV markers requires further approval/analysis.

To assess the involvement of EVs in mediating the transport and uptake of SIGS-derived siRNA, barley leaves were sprayed with CYP3RNA, as previously described [6]. EVs were isolated from apoplastic fluids, and EV RNA cargos were analyzed by RNA-seq. We found that the overall amount of siRNAs that mapped to the sprayed CYP3RNA precursor was very low (Figure 1e). Comparing the RNA-seq data with existing EV-siRNA datasets from CYP3RNA-expressing *Arabidopsis* plants was less informative and reliable, because of the divergent dsRNA origins. While a CYP3RNA transgene leads to the constitutive expression and formation of endogenous dsRNA that can be easily incorporated into intracellular vesicles, exogenously applied dsRNA needs to overcome several cellular barriers before being loaded into EVs. Moreover, the amount of dsRNA continuously decreases after foliar spraying due to degradational and dilutional effects. Consequently, the siRNA quantities that reach the lower unsprayed leaf section for loading into EVs might be reduced compared to prerequisites in HIGS. Given these considerations, it was not surprising that the amount of CYP3RNA spray-derived siRNAs was low. In other words, we found less siRNA in barley EVs than in *Arabidopsis* EVs, which led to a low read coverage (number of reads that overlapped at a certain position of the sequence) compared to *Arabidopsis* EVs (Figure 1e) [5]. Importantly, EV biogenesis, as well as the loading and release mechanisms of EVs’ RNA cargo, may also differ greatly between monocot and dicot plant species, which makes it even harder to compare HIGS and SIGS strategies among two different plant species.

However, besides all of the aforementioned concerns regarding comparability, we found some convincing overlaps. For example, the siRNA pattern demonstrated a bias towards siRNAs that matched the CYP51A fragment in the middle of the CYP3RNA triple construct (Figure 1f), which was also observed for *Arabidopsis* [5]. Further analysis enabled the identification of several of the same siRNAs in both systems, *Arabidopsis*–HIGS and barley–SIGS. Our findings also indicate that the majority of siRNAs are 21 nts in length (Figure 1g) and preferentially begin with an A (Figure 1h), while siRNAs in EVs isolated from transgene-expressing (HIGS) *Arabidopsis* plants begin with an A or U [5]. Interestingly, siRNAs that are not derived from the CYP3RNA precursor preferentially begin with a G (Figure 1h). Based on sRNA-seq data revealing the 5′-identities and lengths of HIGS-derived siRNAs, we can speculate regarding the contributing RNA-binding proteins, insofar as they are known for their specific pathosystem. For barley, we observed a high abundance of siRNAs that were 19 nts in length (Figure 1g). We therefore analyzed the relative abundance of siRNAs of each length in comparison to all identified siRNAs, which we mapped to the precursor to compare the siRNA amounts between both species, and found that barley EVs revealed a second peak for 19 nts siRNAs, which we did not observe in EVs from *Arabidopsis* (Figure 1h,i). This finding—along with previously discovered differences in efficiencies between dsRNA originating from endogenous expression (HIGS) and dsRNA originating from exogenous spray [8]—underlines the mechanistic differences between HIGS and SIGS regarding dsRNA uptake, processing, and transfer. In summary, our current knowledge supports a model of HIGS that involves both plant Dicer-mediated processing of transgene-derived dsRNA into siRNAs and ESCRT-III component-mediated RNA transfer—possibly via EVs. Nevertheless, the process by which EVs traverse the plant–fungal interface remains unknown, while the question of whether *Fg* takes up EVs or siRNA/dsRNA released from EVs prior to uptake remains open. In contrast, sprayed dsRNA is only partially processed by plant Dicers, while unprocessed dsRNA was shown to be taken up by *Fg* [6,7]. Nevertheless, future research must determine whether the loading of unprocessed dsRNA into EVs contributes to SIGS.

Taken together, our data revealed CYP3RNA-derived siRNAs in barley EVs, indicating the uptake, transport and procession of exogenous spray-applied dsRNAs. However, whether the EV-mediated uptake of siRNAs is required to induce SIGS requires further verification regarding the fungal uptake ability of EVs containing siRNAs (and dsRNAs). Moreover, we assume that the fungal uptake of SIGS RNA triggers may depend on the lifestyle of the interacting fungal pathogen. Given this assumption, further research is required to unravel the routes of dsRNAs and siRNAs necessary to determine the strengths and limitations of the SIGS strategy in a pathosystem-specific manner.

## 3. Materials and Methods

### 3.1. Plant Cultivation and CYP3RNA Spray-Application

One hundred and sixty second leaves of barley cv. Golden Promise were harvested from plants grown for 3 weeks under long day conditions (16 h light, 22 °C, 60% humidity). The leaves were transferred to square Petri dishes with 1% agar and divided into two groups. The upper part of the first group was sprayed with CYP3RNA diluted in TE buffer and the second group was sprayed with TE buffer as the mock control, as previously described [6], and incubated for 48 h before EV isolation was performed.

### 3.2. Negative Staining and Transmission Electron Microscopy (TEM)

For TEM, copper formvar-coated 300-mesh electron microscopy grids were glow discharged prior to sample application for 40s. Subsequently, 5 µL of the sample, resuspended in PBS, was applied to the grids. Samples were dabbed using Whatman filter paper and grids were washed three times in 50 µL of 2% uranyl acetate and once with distilled water. Needless staining or fixing solutions, buffers and water were removed using Whatman paper between each step. Finally, the grids were air-dried. Preparations were inspected at 120 kV under zero-loss conditions (ZEISS EM912a/b) and images were recorded at slight underfocus using a cooled 2 k × 2 k slow-scan ccd camera (SharpEye/TRS) and the iTEM software package (Olympus-SIS). Two replicates per sample were invested and at least ten meshes per grid were checked to avoid grid to grid or mesh to mesh variations.

### 3.3. Vesicle Size and Concentration Measurements by Nanoparticle Trafficking Analysis (NTA)

For size and concentration predictions, purified barley EVs were diluted (1:50) with PBS. Subsequently, 500 µL of the vesicle suspension was loaded into Nanosight NS300 (Malvern Panalytical). Five measurements were performed at 25 °C and size, concentration prediction and statistical analysis were performed by the NTA 3.2 Dev Build 3.2.16 software.

### 3.4. Identification of Arabidopsis PEN1 and TET8 Homologs in Barley

The *Arabidopsis thaliana* PEN1 (AT3G11820) and TET8 (AT2G23810) paralogs of *Hordeum vulgare* subsp. vulgare (*Hv*) were predicted based on their amino acid sequences. These were obtained from The *Arabidopsis* Information Resource (tair) (Available online: https://www.arabidopsis.org/ (accessed on 10 February 2021)). Paralogs were forecasted by the NCBI’s protein BLAST service (Available online: https://blast.ncbi.nlm.nih.gov/Blast.cgi (accessed on 10 February 2021)) and the phylogenetic tree was built using ETE 3 [12].

### 3.5. Determine siRNAs Originating from CYP3RNA

Vesicle RNA was isolated using the Single Cell RNA Purification Kit (Norgen Biotek, Thorold, Ca) according to the manufacturer’s instructions for cells growing in suspension. RNA concentrations were determined using the NanoDrop spectrophotometer (Thermo Fisher Scientific, Waltham, MA, USA) and RNA was stored at −80 °C before samples were sent for RNA sequencing. Indexed sRNA libraries were constructed from RNA isolated from vesicles with the TruSeq^®^ Small RNA Library Prep Kit (Illumina, San Diego, CA, USA) according to the manufacturer’s instructions. Indexed sRNA libraries were pooled and sequenced on the Illumina MiSeq Platform (1 × 36 SE) and the sequences were sorted into individual datasets based on the unique indices of each sRNA library. The RNAseq libraries can be accessed on the European Nucleotide Archive (https://www.ebi.ac.uk/ena/browser/home (accessed on 11 February 2021); Accession ID: PRJEB45864). The quality of the datasets was examined with FastQC (version 0.11.9) (https://www.bioinformatics.babraham.ac.uk/projects/fastqc/ (accessed on 11 February 2021)) before and after trimming. The adapters were trimmed using cutadapt (version 2.8) [13]. To filter out bacterial contaminations, kraken2 (version 2.1.1) [14] was used with a database obtained from the MGX metagenomics application [15]. All reads marked as unclassified were considered to be of non-bacterial origin and were used for the subsequent alignment. The trimmed and filtered reads were mapped to the CYP3RNA sequence using bowtie2 (version 2.3.2) [16] to identify siRNAs derived from the precursor dsRNA sequence. The mappings were first converted into bedgraph using bedtools (version 2.26.0) [17] and then to bigwig using bedGraphToBigWig [18]. These files were used for visualization with IGV [19]. Read coverage is defined as the number of reads that match at a certain position in the sequence.

### 3.6. Determine Frequency of Different RNA Species

To determine the RNA species, the reference genome and annotation of *Hordeum vulgare* (IBSC v2—release 47) were downloaded from EnsemblPlants [20]. The adapter trimming of raw reads was carried out with TrimGalore (version 0.6.4) (https://www.bioinformatics.babraham.ac.uk/projects/trim_galore/ (accessed on 12 February 2021)), which used cutadapt (version 2.8) [13]. In this process, all reads that became shorter than 18 nts were filtered out. Afterwards, nucleotides with a phred score below 20 and reads retaining less than 90% of their nucleotides in this process were removed using the FASTQ Quality Filter from the FASTX-toolkit (version 0.0.14) (https://github.com/agordon/fastx_toolkit (accessed on 12 February 2021)). The bacterial contaminations were filtered out as demonstrated in the previous section. The remaining reads were aligned to the reference genome using STAR (version 2.7.3a) [21]. The number of different RNA species was examined in R (version 4.0.2) (R Core Team, 2020) using featureCounts from the package Rsubread (version 2.2.5) [22]. featureCounts was run for each sample using the previously downloaded annotations of *Arabidopsis*. The following RNA types were examined: “lncRNA”, “pre_miRNA”, “mRNA”, “ncRNA_gene”, “rRNA”, “snoRNA”, “snRNA” and “tRNA”. All alignments that could not be assigned to a feature were considered as “not assigned”.

## Data Availability

The RNAseq libraries can be accessed on the European Nucleotide Archive (https://www.ebi.ac.uk/ena/browser/home (accessed on 23 June 2021)); Accession ID: PRJEB45864.
